# Energy Requirement of Patients Undergoing Hemodialysis: A Cross-Sectional Study in Multiple Centers

**DOI:** 10.1155/2020/2054265

**Published:** 2020-03-21

**Authors:** Pei-Yu Wu, Yu-Tong Chen, Te-Chih Wong, Hsi-Hsien Chen, Tzen-Wen Chen, Tso-Hsiao Chen, Yung-Ho Hsu, Sheng-Jeng Peng, Ko-Lin Kuo, Szu-Chun Hung, Shwu-Huey Yang

**Affiliations:** ^1^School of Nutrition and Health Sciences, Taipei Medical University, Taipei, Taiwan; ^2^Department of Nutrition and Health Sciences, Chinese Culture University, Taipei, Taiwan; ^3^Department of Nephrology, Taipei Medical University Hospital, Taipei, Taiwan; ^4^School of Medicine, Taipei Medical University, Taipei, Taiwan; ^5^Department of Nephrology, Wan Fang Medical Center, Taipei, Taiwan; ^6^Division of Nephrology, Department of Internal Medicine, Shuang Ho Hospital, Taipei Medical University, New Taipei City, Taiwan; ^7^Division of Nephrology, Cathay General Hospital, Taipei, Taiwan; ^8^Division of Nephrology, Taipei Tzu-Chi Hospital, New Taipei City, Taiwan; ^9^Nutrition Research Center, Taipei Medical University Hospital, Taipei, Taiwan

## Abstract

**Background:**

Energy requirements must be estimated before nutritional care can be provided for patients undergoing hemodialysis (HD). However, the recommended caloric intake for patients has not been conclusively determined because of insufficiently large sample sizes.

**Method:**

This cross-sectional observational study recruited patients undergoing long-term HD from multiple centers as well as people in the general population without chronic kidney disease. People from both groups were matched by sex and age. Resting energy expenditure (REE) was estimated using an indirect calorimeter. Two commonly used equations for estimating REE and daily energy requirement recommended by the National Kidney Foundation's Kidney Disease Outcomes Quality Initiative (K/DOQI) were chosen.

**Results:**

This study had 154 HD patients and 33 matched HD–control group pairs. Age (*r* = −0.36, *p* < 0.01) and dry body weight after dialysis (*r* = −0.36, *p* < 0.01) and dry body weight after dialysis (

**Conclusions:**

Age and dry body weight are the main factors affecting the energy expenditure of HD patients. Furthermore, predicting the energy expenditure of HD patients by measuring the energy expenditure of their sedentary counterparts in the general population with the same sex, age range, and weight may yield better results than using traditional equations for predicting TEE. In East Asian populations, the TEE values were 32 and 30 kcal/kg dry weight for those aged <65 and ≥65 years, respectively. Future prospective cohort studies with larger sample sizes are needed.

## 1. Introduction

According to annual 2016 statistics from the United States Renal Data System, the prevalence of end-stage renal disease (ESRD) has increased [1]. Despite having declined because of improvements in hemodialysis (HD) and allied treatments, the life expectancy of HD patients is lower than that of the general population; additionally, the mortality of HD patients remains higher than that of patients with cancer or cardiovascular diseases [1].

Notably, medical nutrition therapy can substantially reduce the mortality of HD patients and improve their quality of life [2]. Nutrition care in medical nutrition therapy begins with determining the energy requirement. Inadequate caloric intake is a key cause of protein–energy wasting [3], which can increase the mortality of HD patients [4]. However, the recommended energy requirements determined for HD patients has thus far been inconclusive [5, 6] because of insufficient sample sizes. Specifically, study samples have included approximately 100 patients [6] and few patients of Asian descent [7]. The three countries with the highest ESRD prevalence are in Asia, with Taiwan being the highest and having the greatest number of HD patients. Therefore, this study estimated the energy requirements of HD patients and determined the factors affecting energy requirement.

## 2. Materials and Methods

### 2.1. Participants

**Figure 1:**
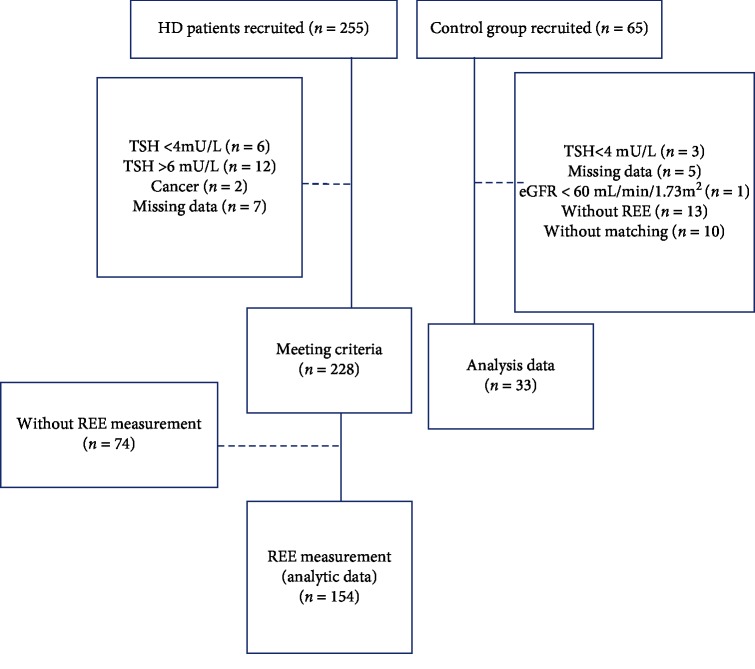
Participant group allocation process. HD: hemodialysis, TSH: thyroid-stimulating hormone, and REE: resting energy expenditure.

HD patients and participants without chronic kidney disease who were aged ≥20 years were recruited and divided into experimental and control groups, respectively. Those who had incomplete data, hyperthyroidism or hypothyroidism, or malignant tumors; who were pregnant; who had undergone an amputation; or who had been hospitalized within 1 month before starting this study were excluded. All participants were recruited from August 2013 to October 2016 and had complete data. The experimental group (*n* = 228) comprised patients who had undergone HD thrice weekly for at least three consecutive months at the dialysis centers of Taipei Medical University Hospital (TMUH), Cathay General Hospital, or Taipei Tzu Chi Hospital; these patients had no notable edema and had adequate dialysis treatment—defined as (dialyzer clearance of urea × dialysis time)/volume of distribution of urea—approximately equal to the patient's Kt/V >1.2. The control group (*n* = 65) comprised outpatients of the department of family medicine at TMUH who had an estimated glomerular filtration rate of >60 mL/min/1.73 m^2^. Both groups were matched by sex and age (±3 years) to form 31 pairs.  Figure 1 depicts how participants were divided. This study was approved by the Institutional Review Boards of TMUH (no. 201302024), Cathay General Hospital (no. CGH-OP104001), and Taipei Tzu Chi Hospital (no. 04-M11−090), and informed consent was obtained from all participants.

### 2.2. Study Design

This was a cross-sectional study. Its methodology was in accordance with that of a previous study [8] and is described as follows. Participants' demographic data, anthropometric measurement data, blood biochemical values, physical activity data, and resting energy expenditure (REE) were collected. The major outcomes were REE and total energy expenditure (TEE), and the potential mediators of REE and TEE were age, sex, body weight (dry body weight after hemodialysis treatment in HD patients), body composition, inflammatory marker, nutrition status, Kt/V, normalized protein equivalent of total nitrogen appearance (nPNA), and status as having hyperglycemia, insulin resistance, hyperparathyroidism, diabetes, or sarcopenia.

### 2.3. Total Energy Expenditure

The REE of all patients was measured. For HD patients, REE was measured on the day of dialysis [5–7]. Patients were instructed to fast for 4 h and not engage in vigorous exercise the day before measurement. REEs were measured using indirect calorimetry (MedGem, Microlife, Golden, CO, USA), which measures oxygen consumption with the constant 0.85 as the respiratory quotient. Steward et al. [9] noted high correlations between REEs measured using a MedGem calorimeter and those measured using a metabolic cart and Douglas bag; they concluded that a MedGem calorimeter yields more accurate assessments of REE and oxygen consumption [9].

The Taiwanese version of the International Physical Activity Questionnaire—Short Form was used to assess 7 days of physical activity, including leisure time as well as work-related and transport-related activities [10]. After data on physical activities were collected, activity factors were estimated in accordance with the recommendations issued by the World Health Organization [11]. TEE was estimated by multiplying the REE by the activity factors and dividing the result by 0.9.

The three energy equations chosen in this study were those introduced by Harris–Benedict [12], Schoenfeld [13], and the National Kidney Foundation's Kidney Disease Outcomes Quality Initiative (K/DOQI) [2] and are presented as follows.

### 2.4. Harris–Benedict Equations

REE for men = 66 + (13.7 × weight in kg) + (5 × height in cm) − (6.8 × age);

REE for women = 655 + (9.6 × weight in kg) + (1.7 × height in cm) − (4.7 × age).

### 2.5. Schoenfeld Equations

The TEEs computed from these two energy equations were estimated using the multiplicative product of the basal metabolic rate and activity factors (Table 1).

### 2.6. K/DOQI-Recommended Equations for HD Patients

TEE for ages <60 years = 35 × dry body weight in kg;

TEE for ages ≥60 years = 30–35 × dry body weight in kg.

### 2.7. Demographics and Anthropometry

Data on the sex, age, Kt/V, and medical history of both groups were obtained through interviews and a review of medical records. In addition, we collected participants' body weights and heights through a chart review. The body weight of HD patients collected in this study was the dry body weight. Experienced nephrologists define dry weight as the lowest body weight at which HD patients feel comfortable after expelling excess fluid (dialysis). The body mass index (BMI) was the body weight (kg) divided by the squared height (m). Participants' body fat percentage and appendicular skeletal muscle mass (ASM) were measured using bioelectrical impedance analysis (BIA) (Inbody s10, Biospace, Seoul, Korea) after they had fasted for at least 4 h, including abstaining from fluids [14, 15]. According to the sarcopenia criteria suggested by the European Working Group on Sarcopenia in Older People, male and female HD patients with ASM/height^2^ values of <7 kg/m^2^ and <5.5 kg/m^2^ are considered to be at risk of sarcopenia [16]. Because all HD patients were stable and had adequate dialysis, nPNA was used as the indicator of dietary intake. According to the recommendation of the K/DOQI, Kt/V was used to calculate nPNA [2].

### 2.8. Blood Biochemical Data

In this study, 8 h fasting and predialysis blood samples were collected and subsequently analyzed in the clinical laboratories of TMUH. The items analyzed included serum creatinine; high-sensitivity C-reactive protein (hs-CRP) (values ≥ 0.3 mg/dL indicated inflammation); albumin (values < 4 g/dL indicated malnutrition); fasting blood glucose (values ≥ 100 mg/dL indicated hyperglycemia; coefficient of variation [CV] = 0.83); insulin (normal range: <25 mIU/L; CV = 4.30); and intact parathyroid hormone (iPTH) (values > 800 pg/mL indicated severe hyperparathyroidism) levels, which were collected through chart review. Serum creatinine level was a surrogate for muscle mass because it was inexpensive to measure and highly correlated with lean body mass when measured using dual-energy X-ray absorptiometry in HD patients [17, 18]. The homeostatic model assessment insulin resistance (HOMA-IR) was calculated in terms of the levels of insulin and fasting blood glucose [19]. Because no recommended level of HOMA-IR in HD patients has been conclusively determined in the literature, this study adopted the median HOMA-IR level as the cutoff. Patients' nutritional status was then determined using the geriatric nutritional risk index (GNRI), which was estimated as follows:

[1.489 × serum albumin (g/dL)] + [41.7 × (dry weight after dialysis/ideal weight)],

where the ideal weight is height (m)^2^ × 22.

Notably, if the weight of HD patients divided by their standard weight was <1, then the ratio was marked as 1. HD patients with GNRI <92 were deemed to be at risk of malnutrition [20].

### 2.9. Statistical Analysis

Data are presented as means ± SD, percentages, medians (range), and 95% confidence intervals (CIs). Factors correlated with the measured energy expenditure were analyzed through their correlations. The factors were as follows: age, sex, dry weight, BMI, diabetes, sarcopenia, serum creatinine, serum insulin, GNRI, Kt/V, and nPNA. Finally, Pearson's correlations were calculated to examine relationships between measured energy expenditure and normally distributed factors, and Spearman's correlations were calculated to analyze relationships between measured energy expenditure and nonnormally distributed factors. The relationships between factors and energy expenditure were determined using a multiple linear regression.

To evaluate the agreement between the measured energy expenditure (obtained through indirect calculations) and predicted energy expenditure (obtained using the equations), this study used Bland–Altman plots and Pitman's test of difference to analyze limits of agreement for each predicted equation, which were stratified by BMI and sex because these factors may influence the agreement. BMI was grouped using 24 kg/m^2^ as the threshold because the accuracy of energy prediction equations is affected by both overweight and obesity [21]; overweight or obesity was defined as BMI ≥ 24 kg/m^2^ according to the 2000 World Health Organization Asian Pacific Guidelines [22]. Bland–Altman plots indicated the error (mean of the individual differences between two methods) and limits of agreement. Pitman's test of difference in variance provides an *r* value and a *p* value, and a *p* value of <0.05 indicates poor agreement between the two values. In addition, the bias of the predicted equations was defined as the predicted energy expenditure minus the measured energy expenditure. The bias is presented with a 95% CI, and a 95% CI that included zero indicated that the predicted equation was unbiased. We also calculated the precision as a percentage, where precision was indicated if the predicted energy expenditure was within the range of 90%–110% of the measured energy expenditure.

The Shapiro–Wilk test was used to assess normality. For continuous and normally distributed data, a *t*-test was used to compare the difference between HD patients and the control group. For continuous and nonnormally distributed data, the Wilcoxon rank-sum test was used to compare data between the two groups. Categorical data were analyzed using a chi-square test. All analyses were conducted using SAS 9.3 (SAS, Cary, NC, USA); *p* < 0.05 indicated statistical significance.

## 3. Results

**Figure 2:**
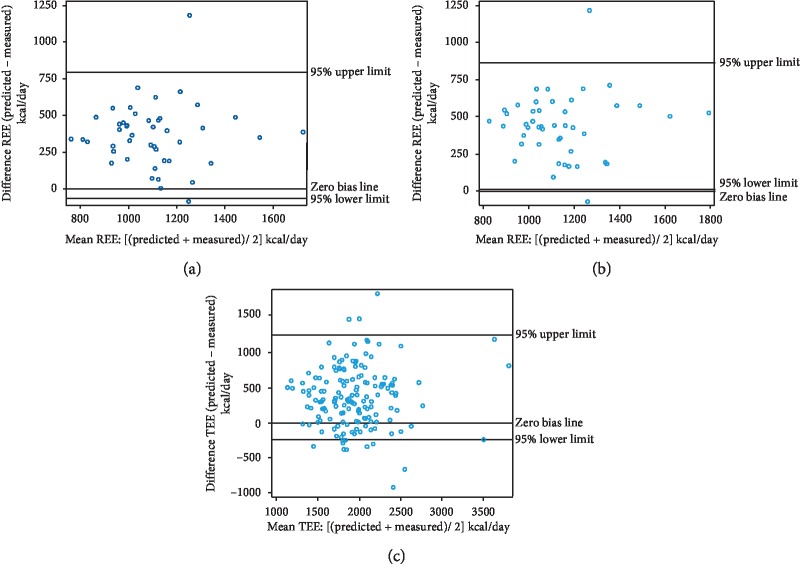
Bland–Altman comparative analysis for predicted energy expenditure (according to the Harris–Benedict, Schoenfeld, and K/DOQI-recommended equations) against measured energy expenditure (measured using indirect calorimetry) in HD patients. REE: resting energy expenditure and TEE: total energy expenditure.(a). Harris–Benedict Equation. (b). Schoenfeld Equation (c). K/DOQI Equation.

In total, 154 HD patients had their REEs measured. Of these patients, 72 (57%) were male. The mean age was 58.9 ± 9.9 years, and the median HD time was 3.8 (range: 0.2–32.3) years (Table 2). Bland–Altman comparative analysis indicated consistency between the predicted energy expenditure and measured energy expenditure (Figure 2).

Regarding the correlation between measured energy expenditure and related factors, measured energy expenditure was positively correlated with sex (male participants had higher energy expenditure), dry body weight, BMI, serum insulin level, and GNRI score, but it was negatively correlated with sarcopenia, Kt/V score, and age (Table 3). Among these factors, dry body weight (*r* = 0.54) and age (*r* = −0.36) were the only significant predictors of measured energy expenditure in multiple linear regression. Multiple linear regression was used to develop better equations for predicting TEE. The regression model of REE included age and dry body weight and had an *R*^2^ of 0.359 (Table 4). In the multiple regression, TEE was significantly related to dry body weight (*β* = 16.6, *p* < 0.001) and age (*β* = −12.1, *p* < 0.001), with an *R*^2^ of 0.355. The regression analysis of the remaining factors all yielded *R*^2^ values of ≤0.355.

According to these results, three commonly used equations with dry body weight and age as variables were chosen, and the differences between the measured energy expenditure and the three equations' estimated energy expenditure are presented in Table 5. For HD patients, regardless of BMI and sex, all equations overestimated energy expenditure and were thus biased in addition to having low precision. For HD patients with BMI ≤24 kg/m^2^, both the Harris–Benedict equation and K/DOQI guidelines equation had good precision. However, for the Harris–Benedict and K/DOQI equations, only 28% and 34% of participants, respectively, had an estimated TEE within 90%–110% of their measured TEE.

Subsequently, 33 HD patients who underwent REE measurement were paired with the same number of counterparts in the control group. REE, TEE, and TEE per kg of dry body weight did not significantly differ between the two groups (Table 6). HD patients had significantly higher levels of serum insulin, higher levels of hs-CRP, and a significantly higher prevalence of diabetes mellitus relative to their control group counterparts (*p* < 0.05).

## 4. Discussion

In this study, although both age and dry body weight affected the energy requirement of HD patients, traditional energy expenditure equations using these variables could not predict actual energy expenditure. Moreover, the energy requirement of HD patients did not significantly differ from that of their control group counterparts, who had the same sex, age range, and level of physical activity. The indirect calorimeter applied in this study used exhaled gas to measure the REE of HD patients. REE results obtained from this instrument were strongly correlated with those from the metabolic cart and Douglas bag [9]. Moreover, because indirect calorimetry is user-friendly and cost-effective [23], it has been extensively used to measure the energy requirement of HD patients [5, 7]. Thus, the indirect calorimeter employed in this study yielded accurate and clinically applicable measurements.

In this study, commonly used energy expenditure equations—the Harris–Benedict equation, Schoenfeld equation, and K/DOQI-recommended equation—overestimated the total energy requirement of HD patients, consistent with the finding of a previous study [5]. This discrepancy is primarily attributable to the difference in study populations. Specifically, age and ethnicity greatly affect body composition, which in turn affects energy expenditure. Furthermore, the Harris–Benedict and Schoenfeld equations were developed by studying young, healthy people of European descent [13, 24], and the K/DOQI-recommended equation was developed by studying African Americans and European Americans [2]. However, participants in this study were people from East Asia with a mean age of 58.9 ± 9.9 years.

The findings suggest that HD patients and individuals in the matched control group did not significantly differ with respect to TEE. This is consistent with previous studies [5, 7, 25]. In addition, age and dry body weight were major factors affecting the energy requirement of HD patients, consistent with K/DOQI clinical practice guidelines [2] and the findings of previous studies [7, 26]. In a previous study, 13 HD patients were asked to consume a specific diet for 92 days to maintain their dry body weight, and these HD patients had similar total energy requirements as did the general population [26]. In addition, compared with a previous study [25], the present study recruited patients with a wider age range and a broader spectrum of medical conditions that included diabetes. Notably, the average energy requirement of this study's HD patients was similar not only to that of their matched counterparts but also to the Taiwanese government's recommended daily energy requirements from Dietary Reference intakes (DRIs) [27], which are 29–31 and 28–29 kcal/kg for Taiwanese people aged 19–70 and ≥ 71 years, respectively.

Previous studies have suggested that other factors affect the energy requirement of HD patients, including muscle mass, thyroid function [28], inflammation [29, 30], poor glycemic control [31], and hyperparathyroidism (iPTH > 700 pg/mL) [6]. The present study excluded HD patients with abnormal thyroid function to reduce the influence of confounding factors. In addition, HD patients in this study were healthier relative to those in previous studies and that may have contributed to the nonsignificant results. Although the sex distribution, mean age, and proportion of patients with diabetes in this study were similar to those of a multiple-center study in Taiwan and a study at the DaVita hemodialysis center in the United States [32, 33], HD patients in this study had better control of blood glucose, and only 12% and 56% had hyperparathyroidism and inflammation, respectively. This study did not report BIA-measured lean body mass because of poor methodological agreement between BIA use and dual-energy X-ray absorptiometry [15]. Therefore, we used serum creatinine as the marker of muscle mass because in addition to being inexpensive and easy to measure, serum creatinine has been demonstrated to be an indicative muscle marker in HD patients [17, 18]. However, serum creatinine level was affected by residual renal function in 14 HD patients in this study who had an HD treatment period of <1 year. This might have resulted in the serum creatinine level having a lower correlation with both REE and TEE relative to its correlation with BMI.

Other limitations of this study are as follows. First, the number of HD patients who had their REE measured still constituted a small sample size (*n* = 154), although this number is much higher than that of previous studies [5–7]. Furthermore, our cross-sectional study did not allow causal inference based on the correlations of TEE with dry body weight (positive correlation) and age (negative correlation) in HD patients. Nonetheless, these correlations were consistent with those reported in a longitudinal study [26]. Third, this study uses sarcopenia criteria for general population but not for HD patients. There are no sarcopenia criteria for HD patients, and criteria for general population are used to calculate the prevalence of sarcopenia in HD patients [34]. Fourth, this study did not investigate other factors that can influence energy requirement, and future studies should include these factors in larger-scale empirical investigations.

Despite these limitations, this study is more informative than other studies and provides an easier method for measuring the total energy requirement of stable HD patients. This study also investigated a larger sample of patients in a wider age range and with a broader spectrum of medical conditions—including diabetes mellitus and malnutrition—which allows its findings to be extrapolated, albeit with caution [7, 26]. Moreover, this study addressed the gap in the literature regarding factors that influence the energy requirement of HD patients of East Asian descent. Thus, the energy requirement of HD patients can likely be accurately determined in terms of their ethnicity, age, dry body weight, and sedentary activity level.

In summary, age and dry body weight were the main factors influencing the energy requirement of HD patients, which was similar to that of patients without kidney failure who were of the same sex and age group who had the same level of physical activity. Therefore, in East Asian populations, the suggested equations of TEE in HD patients is dry weight (kg) *x* c, where *c* = 32 kcal/kg and 30 kcal/kg for patients aged <65 and ≥65, respectively. However, more extensive research is necessary to refine these equations for HD patients.

## Figures and Tables

**Table 1:** 

Age	Men	Women
18–30 years	15.057 × weight in kg + 692.2	14.818 × weight in kg + 486.6
30–60 years	11.472 × weight in kg + 873.1	8.126 × weight in kg + 845.6
>60 years	11.711 × weight in kg + 587.7	9.082 × weight in kg + 658.5

**Table 2:** Demographic, anthropometric, dietary, and biochemical characteristics of hemodialysis patients (*n* = 154)^1^.

Item	HD patients
*Demographics*	
Gender (male (%))	91 (59%)
Age (years)	58.9 ± 9.9
Time on hemodialysis (years)	3.8 (0.2∼32.3)
Kt/V	1.7 ± 0.4
Charlson comorbidity index	1.7 ± 1.4
Diabetes mellitus (*n* (%))	57 (37%)
Cardiovascular disease (*n* (%))	50 (32%)

*Anthropometry*	
Weight (kg)^2^	62.6 ± 13.1
Body mass index (kg/m^2^)^2^	23.6 ± 3.9
Body fat (%)	27.9 ± 9.9
ASM/ht^2^	5.9 ± 0.8
Sarcopenia	42 (27%)

*Biochemical items*	
Albumin (g/dL)	4.1 ± 0.2
Albumin < 4 g/dL (*n* (%))	42 (27%)
Hs-CRP (mg/dL)	0.6 ± 1.2
Hs-CRP < 0.3 mg/dL (*n* (%))	68 (44%)
Glucose (mg/dL)	122.1 ± 51.0
Glucose < 100 mg/dL (*n* (%))	82 (53%)
Insulin (IU/L)	25.9 ± 35.2
Insulin <25 mIU/L (*n* (%))	57 (37%)
iPTH (pg/mL)	387.5 ± 374.4
iPTH > 800 pg/mL (*n* (%))	19 (12%)
GNRI	101.4 ± 5.5
GNRI <92 (*n* (%))	11 (7%)
Creatinine (mg/dL)	11.3 ± 2.0

*Insulin resistance*	
HOMA-IR (units?)	8.6 ± 12.1

*Measured energy requirement* ^3^	
REE (kcal)	1047.7 ± 261.2
TEE (kcal)	1761.7 ± 451.0
TEE/kg (kcal)	28.6 ± 6.4

^1^ Values presented as a mean ± standard deviation, percentage, or median (range).^2^ Dry body weight. .^3^ Measured with indirect calorimetry. ^4^ASM/ht^2^: men<7, women<5.5. ^*∗*^Significant difference between HD participants with and HD participants without REE measured according to an independent *t*-test (*p* < 0.05). ASM: appendicular skeletal muscle mass, HD: hemodialysis, hs-CRP: high-sensitivity C-reactive protein, ht: height, iPTH: intact parathyroid hormone, GNRI: geriatric nutritional risk index, REE: resting energy expenditure, TEE: total energy expenditure, and HOMA-IR: homeostatic model assessment insulin resistance.

**Table 3:** Factors correlated with resting energy expenditure (REE) and total energy expenditure (TEE) in hemodialysis patients.

	REE		TEE	
Item	*r*	*p* value	*r*	*p* value
Dry weight (kg)	0.54	<0.001	0.54	<0.001
BMI (kg/m^2^)	0.38	<0.001	0.37	<0.001
Serum creatinine (mg/dL)	0.28	<0.001	0.29	<0.001
Sex	0.24	<0.001	0.26	<0.001
Insulin (IU)	0.17	0.04	0.18	0.03
GNRI	0.21	0.01	0.23	0.01
Sarcopenia	−0.39	0.002	−0.38	0.002
Age (years)	−0.36	<0.001	−0.36	<0.001
Kt/V	−0.31	<0.001	−0.31	<0.001
nPNA	−0.15	0.228	−0.16	0.212
Diabetes	−0.04	0.773	−0.04	0.745

BMI: body mass index, GNRI: geriatric nutritional risk index, Kt/V: dialysis treatment adequacy (dialyzer clearance of urea × dialysis time)/volume of distribution of urea, approximately equal to a patient's total body water.

**Table 4:** Multiple linear regression analysis for hemodialysis patients using the resting energy expenditure (REE) or total energy expenditure (TEE) as the dependent variable.

Item	Coefficient	95% confidence interval
REE (*R*^2^ = 0.359)		
Age	−7.0	−8.7, −5.2
Dry weight	9.7	8.4, 11.0

TEE (*R*^2^ = 0.355)		
Age	−12.1	−18.0, −6.1
Dry weight	16.6	12.1, 21.1

REE: resting energy expenditure, TEE: total energy expenditure.

**Table 5:** Summary of limits of agreement, Pitman's test of difference, bias, and precision for the predicted energy expenditure (by each equation) and measured energy expenditure (by indirect calorimetry) in hemodialysis patients, stratified by weight category and sex.^1^.

	Category	Limits of agreement (kcal/day)	Pitman's test of difference in variance (r)	Bias (kcal/day)^2^	% precision
Harris–Benedict^3^	All	−444∼1060	−0.21^*∗*^	282 (264, 300)	24
	Men	−444∼1060	−0.22^*∗*^	317 (292, 342)	21
	Women	−99∼571	−0.61^*∗*^	231 (209, 254)	29
	BMI ≤ 24 kg/m^2^	−444∼756	−0.45^*∗*^	242 (221, 263)	28
	Men	−444∼756	−0.47^*∗*^	261 (233, 290)	24
	Women	−99∼528	−0.79^*∗*^	216 (187, 245)	33
	BMI > 24 kg/m^2^	−91∼1060	−0.24	344 (313, 374)	18
	Men	−71∼1060	−0.31	397 (355, 440)	16
	Women	−91∼571	−0.73^*∗*^	257 (222, 292)	22

Schofield^3^	All	−431∼1122	−0.14	367 (348, 386)	14
	Men	−431∼1122	−0.20	423 (396, 449)	10
	Women	−117∼634	−0.64^*∗*^	288 (265, 310)	21
	BMI ≤ 24 kg/m^2^	−431∼780	−0.25^*∗*^	338 (315, 361)	14
	Men	−431∼780	−0.31^*∗*^	382 (350, 415)	7
	Women	−36∼598	−0.76^*∗*^	278 (250, 306)	23
	BMI > 24 kg/m^2^	−117∼1122	−0.18	414 (381, 446)	15
	Men	22∼1122	−0.28	482 (438, 525)	14
	Women	−117∼634	−0.76^*∗*^	304 (266, 343)	17

K/DOQI^4^	All	−920∼1840	0.04	369 (300, 437)	26
	Men	−920∼1840	−0.04	411 (314, 508)	29
	Women	−378∼1137	0.07	307 (216, 399)	22
	BMI ≤ 24 kg/m^2^	−920∼957	−0.34^*∗*^	207 (134, 280)	34
	Men	−920∼957	−0.43^*∗*^	240 (135, 346)	37
	Women	−378∼705	−0.51^*∗*^	162 (64, 261)	30
	BMI > 24 kg/m^2^	−152∼1840	−0.17	574 (515, 729)	13
	Men	−232∼1840	−0.22	660 (505, 816)	16
	Women	41∼1137	−0.21	560 (427, 693)	9

^1^Limits of agreement: range of differences between predicted total energy expenditure (TEE) (by equation) and measured energy expenditure (by indirect calorimetry), Bias: difference between predicted energy expenditure (by equation) and measured energy expenditure (by indirect calorimetry), and % Precision: percentage of cases where the predicted energy expenditure (by equation) was 90%–110% of the measured energy expenditure (by indirect calorimetry).^2^ Mean (95% confidence interval).^3^ Compared with the measured resting energy expenditure (by indirect calorimetry).^4^ Compared with the measured total energy expenditure (by indirect calorimetry). ^*∗*^*p* < 0.05.

**Table 6:** Comparison of demographic, anthropometric, dietary, and biochemical items in hemodialysis (HD) participants and matched non-HD groups with respect to resting energy expenditure (REE) (*n* = 62)^1^.

Item	HD patients with REE measurement (*n* = 33)	Non-HD group (*n* = 33)
*Demographics*		
Sex (male (%))	14 (42%)	
Age (years)	59.7 ± 14.5	59.9 ± 14.6
Charlson comorbidity index	1.6 ± 1.3	0.4 ± 0.6^*∗*^
Diabetes mellitus (*n* (%))	13 (39%)	2 (6%)^*∗*^
Cardiovascular disease (*n* (%))	11 (34%	5 (15%)
*Anthropometry*		
Weight (kg)^2^	62.2 ± 16.6	62.5 ± 12.9
BMI (kg/m^2^)^2^	23.7 ± 4.7	24.0 ± 4.0
Body fat (%)	31.5 ± 11.0	30.7 ± 8.5
*Biochemical item*		
Albumin (g/dL)	4.1 ± 0.3	4.4 ± 0.4^*∗*^
Hs-CRP (mg/dL)	0.5 ± 0.7	0.2 ± 0.3^*∗*^
Glucose (mg/dL)	110.1 ± 40.4	101.5 ± 41.1
Insulin (IU)	28.9 ± 53.4	10.0 ± 7.7^*∗*^
GNRI	99.9 ± 6.3	107.2 ± 6.6^*∗*^
*Insulin resistance*		
HOMA-IR	7.7 ± 13.5	2.7 ± 2.5^*∗*^
*Energy requirement*		
REE (kcal)	987.9 ± 276.1	932.4 ± 226.6
TEE (kcal)	1646.5 ± 460.1	1561.6 ± 373.9
TEE/kg (kcal)	26.8 ± 5.5	25.3 ± 5.1

^1^ Values are presented as the mean ± standard deviation, percentage, or median (range).^2^ Dry body weight (in HD patients only).^*∗*^ Significant difference between groups, according to an independent *t*-test or chi-square test (*p* < 0.05). BMI: body mass index, hs-CRP: high-sensitivity C-reactive protein, GNRI: geriatric nutritional risk index, TEE: total energy expenditure, and HOMA-IR: homeostatic model assessment insulin resistance.

## Data Availability

The data used to support the findings of this study are available from the corresponding author upon request.
